# Knowledge, Attitude, and Practices Regarding Leprosy Among Nurses Employed at a Tertiary Healthcare Centre in Central India: An Epidemiological Study

**DOI:** 10.7759/cureus.75157

**Published:** 2024-12-05

**Authors:** Ankita Srivastava, Keerthika N, Sanjiv Choudhary, Ariharasudhan MV

**Affiliations:** 1 Dermatology, All India Institute of Medical Sciences, Nagpur, Nagpur, IND

**Keywords:** knowledge attitude practices studies, leprosy, national leprosy eradication programme, nurses' attitude, nurses' knowledge, nurses' practice

## Abstract

Introduction: Leprosy is a common infectious disease in India that can lead to nerve damage and disability. There is a dearth of knowledge regarding leprosy not only among the general public but also among healthcare workers. This knowledge gap leads to the generation of stigma and delay in the detection of new cases. Nurses comprise a major proportion of the healthcare community and can also act as opinion leaders and influencers in society. Unfortunately, many times, they are not well-trained regarding leprosy and its clinical manifestations. Lack of adequate scientific knowledge and a positive attitude can hamper the eradication of this disease with life-long consequences on patients’ lives. Therefore, this study was done to assess the knowledge, social attitudes, and practices about leprosy among nurses working at a tertiary care teaching centre.

Methods: This was a cross-sectional study in which nurses working at a tertiary care centre were asked to mark their responses in a questionnaire comprising questions related to various aspects of knowledge, attitude, and practices on leprosy. The data were collected using online Google Forms (Google Inc., Mountainview, CA) and analysed by Stata software version 17 (StataCorp LLC, College Station, TX).

Results: A total of 215 nurses, including 167 (77.67%) females and 48 (22.33%) males, participated in the survey. The age of the participants ranged from 22 to 38 years. Their work experience varied from less than one month to 15 years. Most (180, 83.72%) of the participants knew that leprosy is caused by a bacterium, and 129 (60%) knew that it is predominantly transmitted by the respiratory route. Most (171, 79.53%) of them were also aware that leprosy affects both peripheral nerves and skin. However, less than half (105, 48.84%) of the participants knew about the correct incubation period. Similarly, only 104 (48.37%) participants could correctly answer the question related to the duration of treatment of leprosy. The majority (173, 80.47%) of the participating individuals believed that leprosy is curable. Two hundred and four (94.88%) participants were aware of the National Leprosy Eradication Program (NLEP), and 200 (93%) knew that treatment for leprosy is available free of cost in government hospitals. Only 10 (4.65%) participants strongly agreed to marry a person cured of leprosy, and 13 (6.04%) participants strongly disagreed to shake hands with a leprosy patient.

Conclusion: A considerable proportion of nurses employed at a tertiary care teaching centre are not aware of the correct incubation period, duration of treatment, and mode of transmission of leprosy. Few of them also have a negative attitude towards the disease, which could be detrimental to the patients. It is essential to adequately train them in order to instill the right knowledge and develop the correct perception about leprosy so that they can counsel and care for ailing patients in a healthy manner and move towards the goal of eradication of leprosy.

## Introduction

Leprosy, also known as Hansen’s disease, is still a neglected tropical disease, and almost 2,00,000 new cases are being reported every year globally, of which the majority are from Southeast Asia [1]. India achieved the goal of eliminating leprosy as a public health problem in December 2005, and there was nearly a 97% reduction in patient load as compared to 1982 [2]. But, at the same time, it is to be noted that out of the 127 countries that reported new cases in 2020, India was amongst the three countries with the highest number of new cases [3]. It must be noted that leprosy is a leading cause of peripheral neuropathy, which can lead to permanent disability if not treated timely and adequately [4, 5]. Despite the achievements in reducing the prevalence globally and nationally, there is a dearth of correct scientific knowledge in society regarding leprosy, not only in the general public but also among healthcare workers. The stigma associated with the disease adds to the psychosocial problems faced by the patients. Due to stigma, social consequences of the disease may persist even after the completion of treatment. This cycle of stigma is continued by the lack of knowledge, understanding, and incorrect beliefs [6].

Even in the present era, unfortunately, many of the healthcare workers have inadequate knowledge about leprosy [7-10]. Amongst various healthcare workers, nurses comprise a major proportion, and they also act as opinion leaders in influencing a wider group of the population. Unfortunately, many times, they are not well-trained regarding leprosy and its clinical manifestations. An old study conducted among nurses from Nigeria revealed a below-average basic knowledge and a largely negative perception of leprosy by most of the respondents [11]. A more recent study amongst healthcare workers in the Philippines revealed that only 32 (38%) of participants from the nursing division had high knowledge about leprosy and 56 (67%) had a positive attitude [7]. Previous studies in India also portray the need to hold training at regular intervals among frontline workers to update their knowledge about leprosy [9, 12].

Till now, very few studies from the Indian subcontinent have tried to assess the knowledge and attitude of healthcare workers towards leprosy [9, 12, 13]. However, none of these studies have specifically focused on the nurses. This study was, therefore, conducted to assess the knowledge and attitude about leprosy amongst nurses working at a tertiary care teaching institute. Routine job responsibilities of nurses bring them closer not only to the patient but also to their families. Hence, good scientific knowledge among the nurses about the disease will help in the elimination of stigma and the social inclusion of patients. Thus it is essential for nurses to have the right knowledge and perceptions about leprosy. Also, to effectively participate in disease control, enough knowledge regarding clinical features, mode of transmission, and complications is vital.

## Materials and methods

The study was conducted at the All India Institute of Medical Sciences, Nagpur, at a tertiary care teaching hospital in Central India, and started after obtaining appropriate approvals from the research cell and the Institutional Ethics Committee (approval number: IEC/Pharmac/2023/655; date: 18/10/2023). The study was conducted over a period of six months from November 2023 to April 2024.

Study setting

This study was carried out at a tertiary care teaching centre with various broad speciality and super speciality departments. The participating nurses were posted at various outdoor, indoor, emergency, and critical care units under different departments. 

This was a cross-sectional, questionnaire-based study in which the participating nurses were employed at the tertiary care centre, and they were asked to mark their responses in a well-structured questionnaire comprising questions related to various aspects of knowledge, attitude, and practices on leprosy.

The questionnaire was developed by the investigators after an extensive literature review, taking expert opinion from the faculties of dermatology, community medicine, and the College of Nursing to identify the key areas encompassing various aspects of knowledge, attitude, and practices regarding the disease. To assess the face validity, the questionnaire was initially administered to 20 volunteers among the nurses posted in the outdoor and indoor areas. The questions that lacked clarity or were difficult to comprehend were modified or deleted at this stage. The final questionnaire included a total of 24 questions: five on demographic data, nine based on knowledge domain, and 10 based on attitude and practices (Appendix A). 

Inclusion and exclusion criteria

The study participants included all consenting nurses working under various departments of the institution. The nurses who were not willing to participate in the survey were excluded from the study.

Sample size was calculated using OpenEpi software, version 3 (The OpenEpi Project, Atlanta, GA), considering a finite population size of 490 (total number of nurses working in the centre at the time of study), awareness regarding leprosy amongst nurses as 50%, alpha (α) as 0.05, beta (β) as 0.2 with a 95% confidence interval within an occupation range of ±5%, and a design effect of one; the sample size was found to be 215. Convenience sampling was done to collect the data.

Data collection

**Figure 1:**
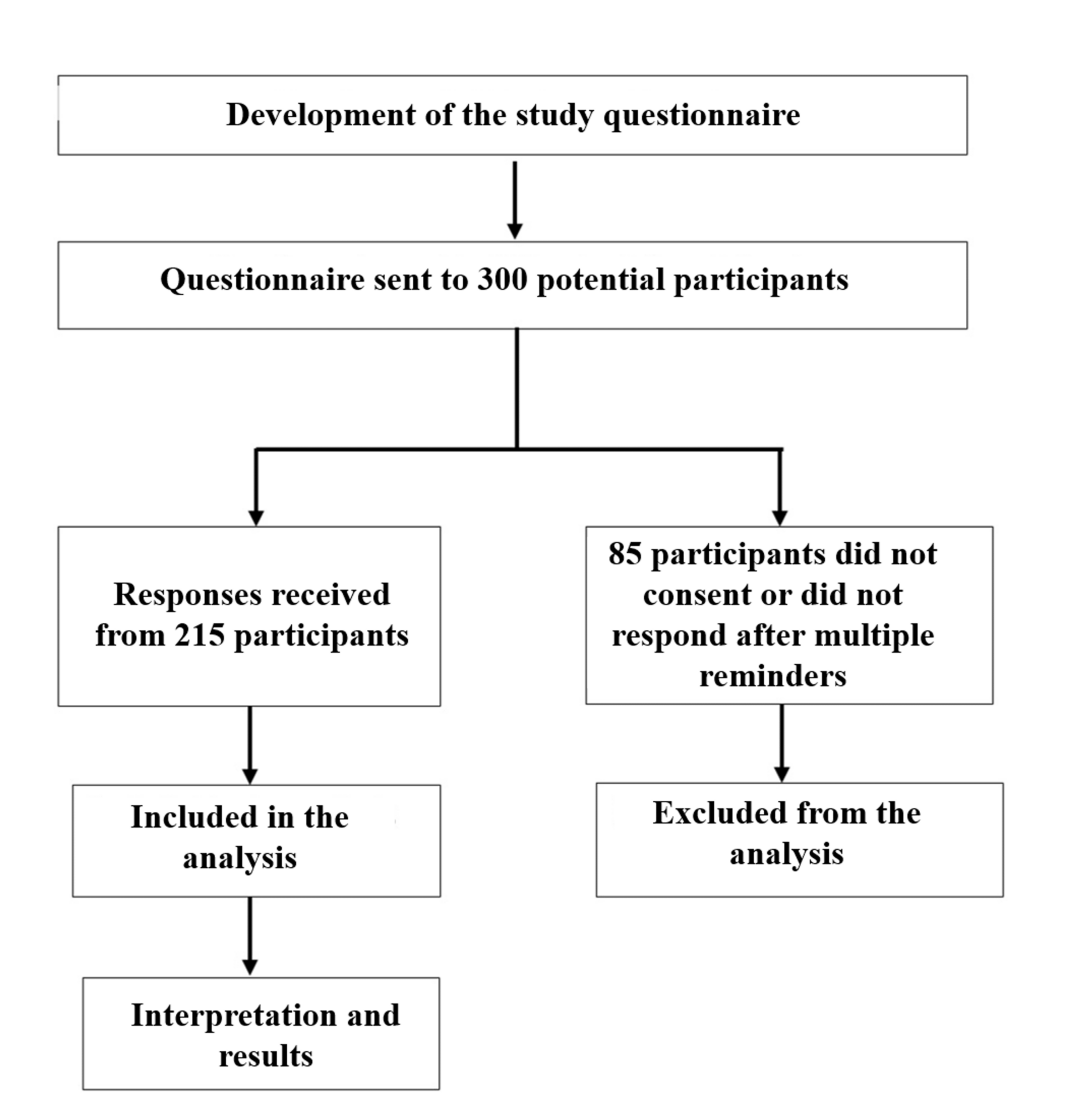
Flowchart depicting the study protocol

The invitation to participate in the study was initially sent by email to 300 nurses working at the centre. All of them were first explained in detail about the purpose of the study. A detailed participant information sheet was given to them, and the questionnaire was shared with those nurses who agreed to participate after obtaining their informed consent. Those nurses who were willing to participate in the survey were sent a link to the Google Form (Google Inc., Mountain View, CA) to their email addresses and were requested to fill out the form and submit it. Two hundred and fifteen participants filled out the Google Form; the remaining 85 invitees either did not give consent to participate or did not respond even after three reminders, hence were excluded from the analysis. As all questions in the Google Form were compulsory, we did not receive any incomplete forms. After the submission of forms, the participants were briefly explained theoretical facts and myths about leprosy in a one-to-one manner in order to enhance their knowledge. The study protocol flowchart is depicted in Figure 1.

Statistical analysis

The data collected from the participants were entered into a Microsoft Excel spreadsheet (Microsoft Corp., Redmond, WA) and analysed by Stata software version 17 (StataCorp LLC, College Station, TX). Descriptive variables were expressed in percentages and proportions. Categorical variables were analysed using the chi-square test with a p-value of < 0.05.

## Results

A total of 215 nurses, including 167 (77.67%) females and 48 (22.33%) males, participated in the survey. The age of the participants ranged from 22 to 38 years (Table 1).

**Table 1:** Age and gender distribution of participants (n = 215)

Age group (in years)	Male	Female	Total	Percentage
21-25	4	48	52	24.19%
26-30	37	107	144	66.98%
31-35	7	11	18	8.37%
36-40	0	1	1	0.47%
Total	48	167	215	100%

The participants' work experience varied from one month to 15 years (Table 2).

**Table 2:** Distribution of participants according to the duration of their work experience (n = 215)

Months of experience	No. of participants	Percentage
0 - 12	51	23.72%
13 - 60	144	66.98%
61-120	17	7.91%
>120	3	1.40%
TOTAL	215	100%

Out of 215 participants, only 99 (46.5%) had encountered cases of leprosy. Around 129 (60%) participants knew that leprosy is predominantly transmitted by the respiratory route. A small number of participants (n=15, 6.98%) believed it to spread through food and water. Around 62 (28.84%) participants responded that it gets predominantly transmitted by touch. It was further noticed that participants with higher work experience were more likely to have correct knowledge regarding the mode of transmission of leprosy. This was statistically significant with p = 0.015 (Table 3).

**Table 3:** Knowledge of participants regarding the mode of transmission of leprosy in relation to their work experience (n = 215)

Leprosy is transmitted mainly by	No. of participants (out of 215) grouped as per the duration of their work experience (in months)				Grand total
	0 -12	13 - 60	61 -120	>120	
Don’t know	3 (5.88%)	6 (4.17%)	0	0	9 (4.19%)
Food and water	6 (11.76%)	9 (6.25%)	0	0	15 (6.98%)
Respiratory route	19 (37.25%)	96 (66.67%)	13 (76.47%)	1 (33.33%)	129 (60%)
Touch	23 (45.10%)	33 (22.92%)	4 (23.53%)	2 (66.67%)	62 (28.84%)
Grand total	51 (100%)	144 (100%)	17 (100%)	3 (100%)	215 (100%)

Most of the participants (n=180, 83.72%) knew that leprosy is caused by a bacterium; however, 30 (13.95%) believed it to be caused by a virus, and five (2.36%) did not know about the cause of leprosy. Not even a single participant considered any devil’s curse as the cause of leprosy. Most of the participants, that is, 171 (79.53%), knew that leprosy affects both peripheral nerves and skin. One hundred and five (n=105, 48.84%) participants responded correctly that the incubation period of leprosy can be as high as five years, while 54 (25.12%) of participants gave 6 months as the answer. When asked about the duration of treatment of multibacillary leprosy, 104 (48.37%) participants answered it to be one year; however, almost an equal number (n=102, 47.44%) of nurses responded six months as the answer for the same question. The majority (n=173, 80.47%) of the participating individuals believed that leprosy is curable. However, the remaining participants (n=42, 19.53%) were of the opinion that leprosy is not curable. The majority (n=204, 94.88%) of the participants were aware of the National Leprosy Eradication Program (NLEP), and around 200 (93%) participants were also aware of the fact that medicine for leprosy is available free of cost in government hospitals. Most (n=206, 95.81%) of the nurses who participated in the study were aware that leprosy can present as a loss of sensation.

Detailed responses of participants to questions regarding attitude and practices are tabulated below in Table 4.

**Table 4:** Responses of participants to questions regarding attitude and practices regarding leprosy (n = 215)

Question	No. of respondents (%)					Grand total
	Strongly agree	Agree	Neutral	Disagree	Strongly disagree	
Persons affected by leprosy shall join social gatherings and religious activities.	40 (18.6%)	110 (51.16%)	38 (17.67%)	19 (8.84%)	8 (3.72%)	215 (100%)
I shall share my work environment with a person affected by leprosy.	37 (17.21)	112 (52.09%)	45 (20.93%)	13 (6.05%)	8 (3.72%)	215 (100%)
I am ready to marry a person who has been cured of leprosy.	10 (4.65%)	62 (28.84%)	98 (45.58%)	32 (14.88%)	13 (6.05%)	215 (100%)
I would avoid sharing a room with a family member diagnosed with leprosy.	16 (7.44%)	48 (22.33%)	72 (33.49)	52 (24.19%)	16 (7.44%)	215 (100%)
If am diagnosed with leprosy I will be comfortable informing my friends and close relatives.	62 (28.84%)	96 (44.65%)	46 (21.40%)	10 (4.65%)	1 (0.47%)	215 (100%)
I shall shake hands with a person affected by leprosy.	29 (13.49%)	98 (45.58%)	50 (23.26%)	25 (11.63%)	13 (6.05%)	215 (100%)
Leprosy is a disease that we should fear.	7 (3.26%)	39 (18.14%)	47 (21.86%)	77 (35.81%)	45 (20.93%)	215 (100%)
We should perform proper handwash before and after dealing with patients suffering from leprosy.	135 (62.79%)	61 (28.37%)	12 (5.58%)	3 (1.40%)	4 (1.86%)	215 (100%)
Mask and gloves are to be worn while taking care of patients suffering from leprosy.	123 (57.21%)	72 (33.49%)	10 (4.65%)	6 (2.79%)	4 (1.86%)	215 (100%)

More than half (n=110, 51.16%) of participants agreed that persons affected by leprosy should join social gatherings, and 112 (52.09%) agreed to share their work environment with persons affected by leprosy. When asked if they were ready to marry a person cured of leprosy, 98 (45.58%) participants responded with the option of neutral, and only 10 (4.65%) strongly agreed.

One-third (n=72, 33.49%) of participants chose to give a neutral answer when asked if they would avoid sharing a room with a family member who is diagnosed with leprosy. Around 96 (44.65%) agreed that they were comfortable in informing their friends and relatives if diagnosed with leprosy. When asked if they should shake hands with a person affected by leprosy, 13 (6.05%) participants strongly disagreed. When asked if leprosy is a disease that we should fear, only seven (3.26%) participants strongly agreed. The majority of the respondents (n=135, 62.79%) strongly opined that they need to perform proper handwashing before and after dealing with a patient suffering from leprosy, while 123 (57.21%) strongly believed that it was essential to use a mask and gloves while taking care of a patient suffering from leprosy. When the participants were asked what they would do if they ever came across a patient with symptoms of leprosy, 138 (64.19%) responded that they would counsel the patient for a check-up at the earliest, and 67 (31.16%) were ready to bring the patient to the nearest health facility. Only one (0.47%) participant responded to stay away from such a case.

## Discussion

This cross-sectional study attempted to assess the level of knowledge, attitude, and practices regarding leprosy among nurses working at a tertiary healthcare centre in India. The study was helpful in identifying the strengths and areas of deficiencies in terms of knowledge and perceptions about a highly stigmatised disease. Although previous studies from India have tried to study these aspects among healthcare workers, none of them has specifically focused on nurses so far [9, 12-14].

In the present study, 167 (77.67%) of respondents were females and the rest were males. It is well-known that nursing has been a female-dominated profession all over the world, and male nurses still face a lot of challenges in their profession [15]. This explains the relatively higher participation of females in our study. On the other hand, in previous studies that have incorporated other cadres of healthcare professionals like medical officers, interns, and undergraduate medical students, the proportion of males had been much higher [9, 12, 14]. In the present study, the age of participants ranged from 21 to 40 years, and the maximum number of participants belonged to the age group of 26 to 30 years. This is quite similar to the study in Ethiopia where the median age of the respondents was 26 years [8]. It implies that participants represent the younger generation, and this might also indicate the positive attitude of young nurses towards participation in the study.

The duration of experience was a significant factor in knowledge regarding the route of transmission of leprosy. The correct answer was more likely to be given by participants with higher experience (p-value = 0.015). This is explained by the fact that people are likely to acquire more knowledge and skills with time. The rest of the demographic characteristics were not found to significantly affect the results (p > 0.05). Amongst the respondents, only 99 (46.5%) had encountered a case of leprosy, which is very close to the findings in the study conducted among medical officers in Andhra Pradesh [12].

More than 80% (180 out of 215) of participants correctly answered that bacteria is the causative organism of leprosy. This is quite similar to the results obtained in the studies conducted in Guyana, Ethiopia, and the Philippines, where the majority of health workers knew that leprosy is caused by bacteria [7, 8, 16]. It was heartening to note that not even a single participant believed leprosy to be caused by any devil or curse. Regarding the route of transmission, in our study, 129 (60%) participants responded that it was predominantly transmitted by the inhalation route, while 62 (28.8%) responded that it was transmitted predominantly by touch. This is in contrast to a recent study from the Philippines where the majority of participants thought that it is transmitted through body fluids, secretions, and open wounds, and only 49 (18.5%) correctly knew that leprosy is transmitted through inhalation [7]. It is highly essential to educate the masses about the correct mode of transmission of leprosy, as fear of contracting the disease by touching the patient (due to lack of knowledge) is likely to generate stigma in society. It is important to note that a significant proportion (86 out of 215, 40%) of participating nurses did not know about the correct mode of transmission of leprosy. Hence it is essential to educate them about the correct facts related to the transmission of disease; otherwise, wrong knowledge will propagate stigma and lack of proper nursing care for patients due to fear of contracting the disease.

When asked if leprosy was curable, a major proportion (n=173, 80.47%) of the participants in the current study agreed that it was curable. This is in contrast to the study conducted in Guyana, where more than half (103, 55.67%) of the respondents did not know that leprosy is a curable disease. The same question was asked of the participants; however, multiple choices were given for answers. In this study, only four (2%) participants believed that leprosy was ‘always’ curable while 48 (26%) opined that it was ‘never’ curable [16].

Most of the participants in our study had good knowledge about certain aspects of the disease, like presentation with loss of sensation (n=206, 95.81%), knowledge about NLEP (n=204, 94.88%), and provision of free medicines for leprosy by the government (n=200, 93%). But, at the same time, many of the participants had inadequate knowledge about the incubation period (n=110, 51.16%) and duration of treatment (n=111, 51.62%). Similarly, in the study conducted on general health workers involved in leprosy control activities at public health facilities in Ethiopia, 372 (62%) participants did not know the duration of treatment [8]. This implies that there is a need to train and retrain healthcare professionals in order to update and maintain their knowledge about leprosy.

In our study, 112 (52.09%) of nurses agreed and 37 (17.21%) strongly agreed to share the work environment with a patient with leprosy. This is in contrast with the study conducted amongst nurses in Nigeria where 76 (27%) of participants disagreed and another 75 (27%) strongly disagreed to work in leprosy hospitals [11]. In the present study, around 110 (51.16%) of participants agreed and 40 (18.6%) strongly agreed that persons affected by leprosy should join social gatherings and religious activities. On the other hand, 270 (45%) of the respondents in a previous study agreed that it would be better to isolate persons affected by leprosy from other individuals [8]. A similar opinion about the isolation of patients was noted in another study conducted on nurses [11]. In the current study, only seven (3.26%) of the participants strongly agreed that leprosy is a disease that we should fear. However, in a previous study, 96 (52%) of the respondents were afraid of leprosy [16]. In another study, 180 (65%) of the nurses opined that extra allowances must be provided for accepting the risk of working in leprosy hospitals [11]. Our findings suggest that participants of our study have a much better attitude towards leprosy than in the past. This might be possible because of correct scientific knowledge about the disease. It is essential to educate healthcare providers in detail about leprosy, how it spreads, and its curable nature. If the health care providers themselves are scared of disease, the patients will not only face stigma but will also be neglected during routine medical care.

In our study, we asked the respondents if they were ready to marry a patient who was cured of leprosy. To this question, 98 (45.58%) replied neutral, 32 (14.88%) disagreed, and 13 (6.05%) strongly disagreed. A previous study in Nigeria also revealed that 69 participants (25%) agreed and 121 (44%) strongly agreed that it would be difficult to find a suitable husband for a spinster nurse if they worked for leprosy patients [11]. This implies that even after several years of elimination of leprosy as a public health problem, there is still some social stigma prevalent, even among healthcare professionals like nurses.

In our study, the majority of the participants agreed that gloves and masks have to be used while examining patients suffering from leprosy. The use of a mask probably reflects knowledge of the respiratory route as the predominant mode of transmission, whereas the use of gloves is required while caring for open wounds and/or trophic ulcers. The majority of the participants also agreed to wash their hands before and after dealing with patients with leprosy. These measures do not necessarily reflect stigma but are essential infection control practices.

Limitations of the present study include participation from a single tertiary care centre; hence, the findings cannot be extrapolated across the country. Also, the knowledge and perception about leprosy amongst nurses can be affected by several factors like their qualifications, experience, nature of job profile, type of health care centre, regional difference in prevalence of leprosy, etc. Hence, a multicentric study involving nurses from health care centres from all parts of the country is recommended in order to better identify the deficits in knowledge and attitude towards leprosy in the current era.

## Conclusions

The present study reveals a sufficiently good level of knowledge and a positive attitude about leprosy amongst nurses in a tertiary care centre in India. However, a considerable proportion of nurses were not aware of the correct incubation period, duration of treatment, and mode of transmission of leprosy. Some level of stigma does exist even amongst nurses, especially in personal issues like marriage. It is, therefore, recommended to educate and reinforce the correct knowledge through the conduction of in-service training programs. Although leprosy is already included in the curriculum of nurses, we recommend a special focus on this disease in order to enhance the knowledge of future nurses and to ensure the success of NLEP in India.

## Appendices

Appendix A

Study Questionnaire

Demographical data:

1.     Age:

2.     Sex: M/F/Other

3.     Years of experience:

4.     Department where you are posted:

5.     Have you encountered someone with leprosy till now: Yes/No

Instruction: Please choose the best response for question number 1 to 10

1.  Leprosy is caused by:

(a) Bacteria

(b) Virus

(c) Curse

(d) Don’t know

2. Leprosy is transmitted mainly by:

(a) Touch

(b) Respiratory route

(c) Food and water

(d) Don’t know

3. What parts of the body are commonly affected by leprosy?

(a) Peripheral nerves

(b) Skin

(c) Central nervous system

(d) Both a & b

4. Incubation period of leprosy can be as high as:

(a) 2 years

(b) 5 years

(c) 6 months

(d) 1 month

5. Duration of treatment in multibacillary leprosy is:

(a) 6 months

(b) 1 month

(c) 1 year

(d) 3 months

6. Is leprosy curable?

(a) Yes

(b) No

7. Are you aware of the National Leprosy Eradication Program?

(a) Yes

(b) No

8. Is medicine for leprosy available free of cost at government hospitals?

(a) Yes

(b) No

9. Can leprosy present as loss of sensation?

(a) Yes

(b) No

10. What would you do if you came across a person in your neighbourhood with symptoms suggestive of leprosy?

(a) Counsel patient for check-up at the earliest

(b) Prescribe MDT for leprosy

(c) Bring him/her to the nearby health facility

(d) Stay away from him/her completely

(e) No intervention

Instruction: Kindly mark your answers to the next nine statements in relation to leprosy in the following format:

(a) Strongly agree

(b) Agree

(c) Neutral

(d) Disagree

(e) Strongly disagree

(1) Persons affected by leprosy shall join social gatherings and religious activities

(2) I shall share my work environment with a person affected by leprosy

(3) I am ready to marry a person who has been cured of leprosy

(4) I would avoid sharing a room with my family member diagnosed with leprosy

(5) If I am diagnosed with leprosy I will be comfortable informing my friends and close relatives

(6) I shall shake hands with a person affected by leprosy

(7) Leprosy is a disease that we should fear

(8) We shall perform proper hand washing before and after dealing with patients suffering from leprosy

(9) Masks and gloves are to be worn while taking care of patients suffering from leprosy
